# Blockade of dengue virus entry into myeloid cells by endocytic inhibitors in the presence or absence of antibodies

**DOI:** 10.1371/journal.pntd.0006685

**Published:** 2018-08-09

**Authors:** Ana C. Carro, Luana E. Piccini, Elsa B. Damonte

**Affiliations:** 1 Laboratory of Virology, Department of Biological Chemistry, Faculty of Sciences, University of Buenos Aires, Buenos Aires, Argentina; 2 IQUIBICEN, National Research Council (CONICET)-Department of Biological Chemistry, University of Buenos Aires, Ciudad Universitaria, Buenos Aires, Argentina; Louisiana State University, UNITED STATES

## Abstract

**Background:**

Dengue is the most prevalent arthropod-borne viral human disease in tropical and subtropical regions, caused by four dengue virus (DENV) serotypes. In spite of the increasing global incidence, no specific antiviral therapy is available. Cells of the mononuclear phagocyte lineage are the main targets either for direct antibody (Ab)-independent or Ab-mediated human DENV infection, usually associated to the severe forms of disease. Since the virus entry may be a convenient therapeutic alternative, this study aimed to investigate the mode of DENV internalization into myeloid cells in the absence and presence of DENV Ab and evaluate the inhibitory activity of diverse biochemical inhibitors of endocytosis.

**Methodology/principal findings:**

By infectivity assays and quantitative RT-PCR determinations, it was demonstrated that DENV-2 entry into U937 and K562 cells in the absence of Ab was highly inhibited by the early treatment with ammonium chloride, chlorpromazine and dynasore, but it was not affected by methyl-β-cyclodextrin, indicating that DENV-2 utilizes a low pH-dependent, clathrin- and dynamin-mediated endocytic infectious pathway for the direct entry into both human myeloid cells. To study the Ab-mediated entry of DENV, the experimental conditions for enhancement of infection were established by inoculating immune complexes formed with DENV-2 and the Ab 2H2 or 3H5. The internalization of DENV-2-2H2 or DENV-2-3H5 complexes in both myeloid cells was also dependent on acid pH and dynamin but a differential requirement of the clathrin-mediated endocytic route was observed depending on the FcγR involved in the complex uptake: the infection through FcγRII was dependent on clathrin-coated vesicles whereas the internalization pathway mediated by FcγRI was independent of clathrin. This property was not serotype-specific.

**Conclusions/significance:**

DENV entry into myeloid cells in the absence or presence of Ab can be blocked by diverse biochemical inhibitors affecting the cellular factors involved in endocytosis. The identification of the virus-host interactions involved in virus penetration may allow the finding of host-targeted antivirals widely active against diverse pathogenic flaviviruses with similar requirements for virus entry.

## Author summary

Dengue is currently a widespread viral disease transmitted to human by mosquitoes, with very high prevalence in tropical and subtropical regions of América and Asia. Approximately 2.5 billion people are living in endemic areas and it is estimated that 350 million apparent and inapparent infections occur each year. There is no specific antiviral for treatment of dengue patients. On this basis, the search of antiviral agents is an urgent need. Dengue virus (DENV) entry is an attractive alternative for chemotherapeutic intervention since it represents a barrier to block the beginning of infection. We intended to evaluate the antiviral activity of diverse biochemical inhibitors of endocytosis against DENV in human myeloid cells. Results showed that DENV entry into these cells can be blocked by diverse biochemical inhibitors affecting the cellular factors involved in endocytosis such as intravesicular pH, clathrin-coated vesicles and dynamin, although the presence of non neutralizing antibodies, as can occur in secondary human infections, may alter the entry pathway under certain conditions. Then, the blockade of virus entry with host-targeted inhibitors may be a promising strategy for the development of safe antiviral agents with wide spectrum of activity against DENV and other related pathogenic flaviviruses.

## Introduction

Dengue is currently the most prevalent arthropod-borne viral disease transmitted to human through infected mosquitoes of the genus *Aedes* with about 350 million infections estimated to occur each year [1,2]. Clinical manifestations range from an asymptomatic infection or mild febrile illness, known as dengue fever (DF), to the severe forms leading to dengue hemorrhagic fever and dengue shock syndrome (DHF/DSS) responsible of 25,000 annual deaths [3,4]. There are four serotypes of dengue virus (DENV), DENV-1 to DENV-4, which co-circulate worldwide and give rise to sequential epidemic outbreaks. The initial infection with one DENV serotype leads to lifelong protection against homologous reinfection, but the secondary infection with a heterologous serotype is considered a risk factor for developing severe dengue associated to the phenomenon known as antibody-dependent enhancement (ADE) [3,5]. In this process, antibodies (Ab) elicited by the primary infection bind to the heterotypic virus without infectivity neutralization, and these immune complexes enter into Fcγ-receptor (FcγR) positive cells leading to an increase in DENV replication and pathogenesis [6]. A similar phenomenon may be caused by low levels of homologous Ab and both conditions have been shown to correlate with severe disease outcome, particularly in infants provided with maternal Ab [7,8].

The virus particle contains a single-stranded positive sense RNA included in an inner nucleocapsid and covered by a lipid envelope. The genome codes for a single polyprotein that is cleaved by cellular and viral proteases into three structural proteins (the capsid protein C, which binds to the RNA to form the nucleocapsid; a membrane protein, which matures from the precursor prM; the envelope glycoprotein E, main mediator of virus entry into the cell) and seven nonstructural proteins (NS1, NS2A, NS2B, NS3, NS4A, NS4B and NS5).

Despite the global reemergence and severity of dengue disease, no specific antiviral therapy is available for DENV patients. Then, the search for agents able to block primary DENV infection as well as ADE-related infection is a real need. The blockade of virus entry is a valuable antiviral strategy because an initial barrier may be established to suppress infection. The DENV primary entry is triggered by a wide range of proposed host receptors, such as heparan sulfate, C-type lectins, heat shock protein 70/90, phosphatidylserine receptors and other molecules, that bind to the E glycoprotein [9,10]. After binding, DENV penetrates into the cell by receptor mediated endocytosis and finally the virion uncoating is triggered by the acid pH of the endocytic vesicle through fusion of the viral envelope and the endosomal membrane. In ADE-dependent infection, the viral entry appears to be initiated by binding of Ab to FcγR-bearing cells but it was postulated a possible additional requirement of other primary cellular receptors for the completion of DENV-2 entry [11], and, after this interaction, the precise mode of Ab-mediated virus internalization into the cell is not fully elucidated.

Among the variety of cells susceptible to be infected by DENV, cells of the mononuclear phagocyte lineage, like monocytes, macrophages and dendritic cells, are considered the main targets for DENV human natural infection [5]. The process of DENV entry into different types of mammalian epithelial cells has been found to be controlled by various cell- and virus serotype/strain-dependent factors with several alternative routes for internalization and trafficking inside the cell until the nucleocapsid release is detected in the cytoplasm [12–20]. By contrast, very few studies were performed about the direct DENV entry into human myeloid cells [21–23]. A recent study performed in murine macrophage P388D1 cells showed that the entry of DENV-2 in the absence and presence of Ab was caveola-independent, dynamin and pH-mediated and a variable role of clathrin, actin and PI3K as well as Rab-GTPases was detected on the virus entry pathway depending upon the participation of Ab [24].

The present study intended to investigate the infectious entry route of DENV in human myeloid cells in the absence and presence of antibodies by using pharmacological inhibitors of diverse endocytic pathways. The identification of the specific interactions for viral penetration in both the direct Ab-independent and the Ab-mediated infection of human cells may be useful for the development of antiviral therapies targeted to block the early events of the DENV multiplication cycle in the natural infection.

## Methods

### Cells and viruses

The human myelomonocytic cell line U937 and the human myelogenous erythroleukemic K562 cells (both provided by Dr. R. Gamberale, Academia Nacional de Medicina, Buenos Aires, Argentina) were grown in RPMI medium 1640 supplemented with 10% fetal bovine serum (FBS) and 50 μg/ml gentamycin. Medium was supplemented with HCl/NaHCO_3_ when incubated at 37°C under 5% CO_2_. The C6/36 mosquito cell line from *Aedes albopictus*, adapted to grow at 33°C, was cultured in L-15 medium (Leibovitz) supplemented with 0.3% tryptose phosphate broth, 0.02% glutamine, 1% MEM non-essential amino acids solution and 10% FBS. Vero (African green monkey kidney) cells were grown in Eagle's minimum essential medium (MEM) supplemented with 5% FBS. For maintenance medium (MM), the serum concentration was reduced to 1.5%.

DENV-2 strain NGC and DENV-3 strain H87 were propagated in C6/36 cells and viral titers were determined by a standard plaque assay in Vero cells as described previously [25].

### Antibodies and reagents

The mouse anti-DENV monoclonal Ab 3H5 produced by the hybridoma HB46 (ATCC, USA), kindly supplied by Dr. Irene Bosch, University of Massachusetts Medical School, USA, and 2H2 (Chemicon, USA) were used. 3H5 is an IgG1 Ab that reacts with DENV-2 E protein and binds to IgG Fc receptor II (FcγRII) whereas 2H2 is an IgG2a Ab reactive with the prM protein of all members of DENV complex and binds to both FcγRI and FcγRII [26]. The clone AT10 of an anti-human FcγRII Ab was provided by Dr. Mirta Giordano (Academia Nacional de Medicina, Buenos Aires, Argentina).

TRITC-human transferrin was from Molecular Probes (USA) and FITC-cholera toxin B subunit was purchased from Sigma-Aldrich (USA). Chlorpromazine, dansylcadaverine, ammonium chloride, β-methylcyclodextrin, dynasore, and acridine orange were purchased from Sigma-Aldrich (USA).

### Cell viability

To evaluate the effect of the inhibitors on cell viability, U937 and K562 cells were incubated with medium containing 2-fold serial dilutions of each compound. After 3 h of incubation at 37°C, cell viability was determined by staining with 0.4% trypan blue and the number of trypan blue positive and negative cells was counted in a hemocytometer under light microscope.

### Control of the biochemical inhibitor functions

To assess the effectiveness of drug treatment on myeloid cells, different specific markers of the compound activity were used. Cells were treated or not with the inhibitors as in the virus infection assay and then incubated with 15 μg/ml TRITC-transferrin (for chlorpromazine and dynasore), 0.3 μg/ml FITC-cholera toxin B subunit (for methyl-β-cyclodextrin) or 1 μg/ml acridine orange (for ammonium chloride) for 1 h at 37°C. Then, the cells were processed for visualization in a fluorescence microscope Olympus BX51.

### Effect of drug treatment on virion viability

DENV-2 suspensions containing 1x10^6^ PFU/ml were incubated with an equal volume of MM with or without different concentrations of compound for 2 h at 37°C. Then, samples of control and treated virions were chilled and filtered through cellulose membranes (Vivacon 500, 100,000 MWCO, Sartorius) to eliminate free drug. The residual infectivity was determined by plaque formation.

### Effect of drug treatment on DENV infection by infectivity assay

Cultures of U937 and K562 cells were pretreated for 1 h at 37°C with the non-cytotoxic concentrations of each inhibitor in MM or in MEM without serum for the cholesterol-reactive compound methyl-β-cyclodextrin. Then, the cells were infected with DENV at a m.o.i. of 5 PFU/cell or with the corresponding mixtures DENV-Ab in the presence of the drug, except for dynasore and methyl-β-cyclodextrin where drug-pretreated cultures were extensively washed before infection to eliminate the compound and were further incubated in the absence of compound. After 2 h at 37°C the virus inocula were removed, then the cultures were washed with PBS and further incubated at 37°C in MM without inhibitor. These experimental conditions assessed that the effect of the compound was exerted only during the initial entry process. Supernatants were collected after 48 h or 72 h of infection in the absence or in the presence of Ab, respectively, to evaluate the extracellular virus yields by plaque formation in Vero cells.

To confirm that the effect of each drug was restricted to the virus entry process, another set of cultures was infected with DENV-2 at a m.o.i. of 5 PFU/cell and incubated for 2 h at 37°C. Cells were then washed with PBS and incubated with MM containing each compound during 2 h at 37°C. After washing the cells, MM without compound was added. At 48 h p.i. the extracellular virus yield was determined by plaque formation in Vero cells.

### Effect of drug treatment on DENV infection by quantitative RT-PCR

Cultures containing 3x10^5^ U937 or K562 cells were pretreated for 1 h at 37°C with 50 mM ammonium chloride, 20 μM chlorpromazine, 160 μM dynasore or 2.5 mM methyl-β-cyclodextrin. Then, the cells were infected with DENV-2 at a m.o.i. of 5 PFU/cell in the presence of the drug, except for dynasore and methyl-β-cyclodextrin where the drug-pretreated cultures were extensively washed before infection and were further incubated in the absence of compound. After 2 h at 37°C the virus inocula were removed, then the cultures were washed with PBS and incubated at 37°C in MM without inhibitor. At 12 h p.i. the cells were washed thrice with 1 ml PBS and then total RNA was extracted from cells by using TRIzol (Invitrogen, USA) according to the manufacturer’s instructions. For quantification of viral RNA, a quantitative RT-PCR assay was conducted using Taq Man technology and primers and probe targeted to amplify nucleotides 10,419 to 10,493 within the viral 3′UTR as previously described [25]. Briefly, each 25 μl reaction mix contained 10 μl of extracted RNA sample and final concentrations of 1× RT-PCR buffer (10 mM Tris–HCl pH 8.4, 50 mM KCl, 0.01% w/v gelatin, and 10 mM DTT), 2.5 mM MgCl_2_, 250 μM deoxynucleoside triphosphates, 100 nM primer 5′ (5′-CCTGTAGCTCCACCTGAGAAG-3′), 100 nM primer 3′ (5′-CACTACGCCATGCGTACAGC-3′), 100 nM probe (5′-/56-FAM/CCGGGAGGCCACAAACCATGG/36-TAM/-3′), and 100 units M-MLV RT (Promega). Reverse transcription was allowed to proceed for 1 h at 37°C, and then 2 units of Taq DNA polymerase (Invitrogen, USA) were added to each reaction tube. PCR amplification and detection were performed using the following conditions: 95°C for 3 min (1 cycle), and then 40 cycles of 95°C for 15 s and 61°C for 1 min. A standard curve was generated using in vitro transcribed DENV RNA replicon [25].

### Establishment of ADE conditions

Serial dilutions of DENV-specific Ab or media (control without Ab) were incubated with 1.5 x 10^5^ PFU of DENV-2 or DENV-3 for 1 h at 37°C. Then, cultures of U937 or K562 cells grown in 24-well microplates were infected with the virus-Ab mixtures and incubated at 37°C. Supernatant samples of each infected culture were titrated by PFU at 72 h p.i. Also control experiments of receptor blockade were performed by incubating the cells for 30 min at 4°C, previously to the addition of the virus-Ab mixtures, with: a) soluble human IgG centrifuged at 15000 g for 30 min to block FcγRI; b) human IgG aggregated by heating at 62°C for 15 min or the clone AT10 of an anti-human FcγRII monoclonal Ab, both conditions to prevent ADE by FcγRII blockade.

### Statistical analyses

Statistical analyses were performed using GraphPad Prism software. Comparison of means was tested by Student’s unpaired t-test or ANOVA analysis and Dunnett’s multiple comparison post hoc test, using data obtained from three independent experiments. Statistical significance is depicted in figures.

## Results

### The effect of the endocytic inhibitors on the DENV entry into myeloid cells

We used two human myeloid cell lines bearing Ab-binding Fcγ-receptors to analyze comparatively the entry route of DENV in the absence and presence of Ab. The myelomonocytic U937 cells express both the high affinity Fcγ-RI (CD64) and the low affinity Fcγ-RII (CD32) receptors whereas the myelogenous erythroleukemic K562 cells express only Fcγ-RII, and both lines were reported to support in vitro ADE of DENV [23,27,28]. Several pharmacological inhibitors affecting different endocytic pathways were employed: ammonium chloride, a lysosomotropic weak base that immediately raises the pH of acidic vesicles, was used to test pH-dependence [29]; chlorpromazine was tested as a known inhibitor of clathrin-mediated endocytosis [30]; methyl-β-cyclodextrin is an sterol-binding drug that sequesters cholesterol by extraction of this lipid from the membranes affecting the lipid rafts/caveolae dependent pathways [31,32]; and the participation of dynamin was evaluated with dynasore, an inhibitor of dynamin GTPase activity [33].

**Fig 1:**
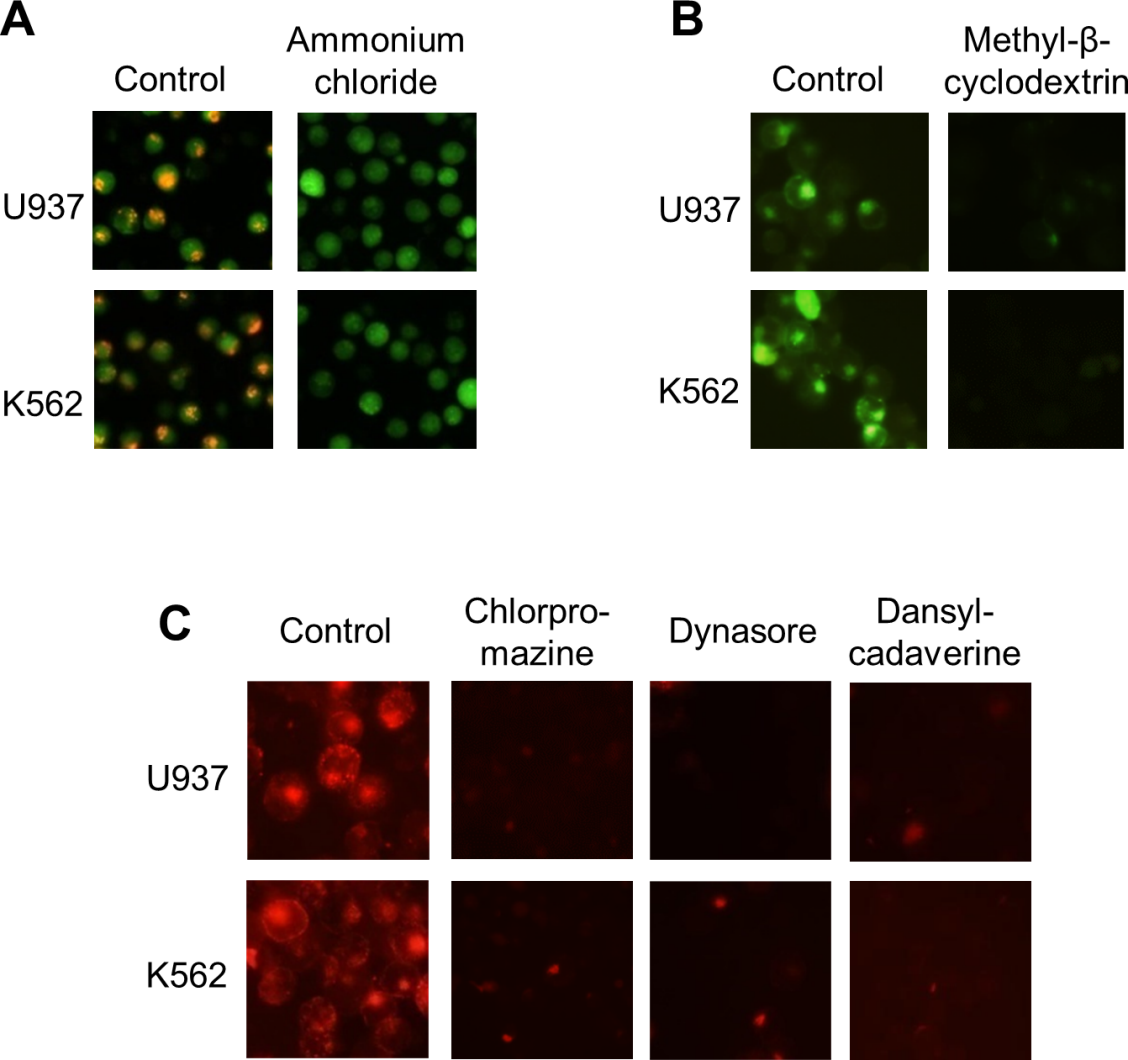
Control of the endocytosis inhibitors function. U937 and K562 cells were treated with 50 mM ammonium chloride (A), 2.5 mM methyl-β-cyclodextrin (B), 20 μM chlorpromazine, 150 μM dansylcadaverine, 160 μM dynasore (C) or untreated (control) and then incubated with acridine orange (A), FITC-labelled cholera toxin (B) or TRITC-labelled transferrin (C). Controls without drugs were performed and the samples were visualized by fluorescence microscope.

The noncytotoxic conditions of each drug for early treatment during virus entry in both human cell lines were initially assessed by trypan blue staining (S1 Fig). Furthermore, the inhibitory activity of the compounds on endocytic compartments and pathways was corroborated with specific markers, like acridine orange staining, labelled transferrin and cholera toxin. A total blockade in vacuolar acidification as well as in transferrin and cholera toxin uptake was observed after treatment with the corresponding inhibitor (Fig 1A–1C).

Next, the effect of endocytic inhibitors on virion viability was studied to assess that any inhibitory action observed in DENV infected cells would be due to their effect on the cellular processes involved in virus infection. Virion suspensions of DENV-2 were incubated with each inhibitor and, after separation of the free compound through cellulose membranes, the remaining infectivity was determined by a plaque assay. A dose-dependent decrease of DENV-2 viability was observed after virion incubation with dynasore (S2 Fig) whereas no virus reduction in virus titers was detected with the other inhibitors. A similar DENV-2 inactivating activity has been previously described for the cholesterol reactive compound methyl-β-cyclodextrin [34].

Thereafter, the effect of the inhibitors on virus production from infected cells was studied by cell pretreatment with the concentrations of the inhibitors chosen according to the viability and endocytic activity data: ammonium chloride (12.5–50 mM), chlorpromazine (10–30 μM), methyl-β-cyclodextrin (1.25–2.5 mM) and dynasore (40–160 μM). After pretreatment, cells were infected with DENV-2 at a m.o.i. of 5 PFU/cell, adjusted to produce a level of approximately 10^5^−10^6^ PFU in the supernatant of K562 and U937 cells at 2–4 days after infection, since at m.o.i. in the range 0.1–1 the level of virus production was very low or undetectable (S3 Fig). To evaluate the inhibitory effect on the virus entry and avoid potential pleiotropic effects, the compounds were present only during the initial 2 h of infection, except for dynasore and methyl-β-cyclodextrin where only drug pretreatment was performed to avoid virus inactivation by direct contact between drug and virions. In all cases, the effects of the early drug treatment were determined by virus yield titration at 48 h p.i.

**Fig 2:**
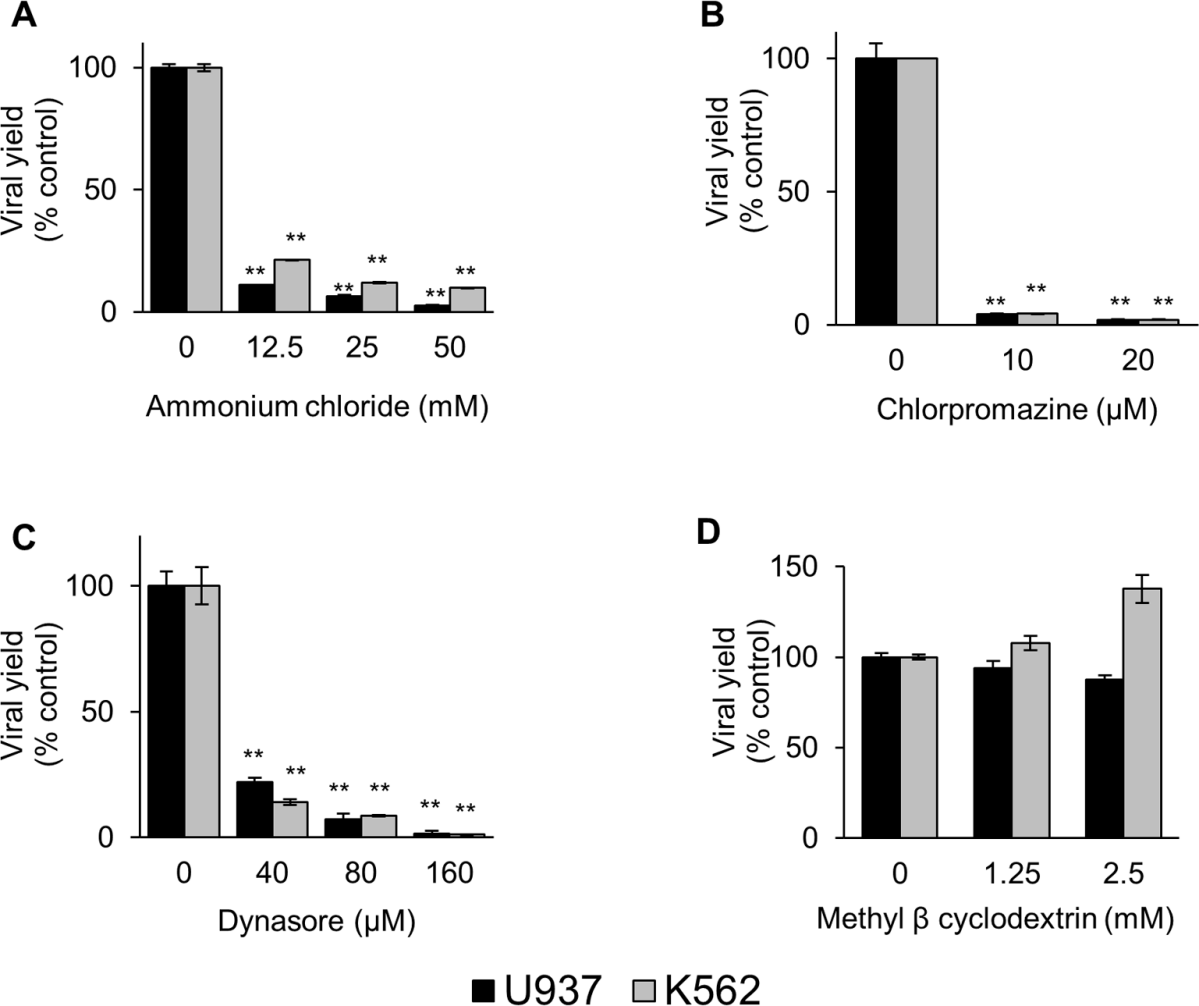
Effect of the endocytosis inhibitors on DENV-2 infective virus production in U937 and K562 cells. U937 (black bars) or K562 (grey bars) cells were treated with ammonium chloride (A), chlorpromazine (B), dynasore (C) or methyl-β-cyclodextrin (D), and then infected with DENV-2. At 48 h p.i., the virus yields were determined by plaque formation in Vero cells and the results are expressed as % of virus multiplication with respect to a control of infected cells without drug treatment. Each bar is the mean of three independent experiments ± SEM. Asterisks indicate statistical significance between treated and control infected cells (** P < 0.0001).

As expected and in accordance with the structural studies about the low pH triggered-E glycoprotein conformational changes required for membrane fusion [35–37], a high reduction in virus yield was observed in cells treated with ammonium chloride (Fig 2A), when the acid pH of intracellular vesicles was neutralized (Fig 1A). The pH-dependent entry of DENV-2 into myeloid cells suggests that the virus is internalized by receptor-mediated endocytosis and reaches an endosomal compartment where the fusion occurs.

Next, the requirement of clathrin for endocytosis was evaluated by using chlorpromazine. A drastic inhibition in infective virus production was observed in both cells treated with chlorpromazine at noncytotoxic concentrations (Fig 2B), under treatment conditions that effectively blocked the internalization of TRITC-labelled transferrin (Fig 1C). The endocytic pathway for DENV-2 entry into U937 and K562 cells was further characterized by testing the participation of dynamin, a GTPase essential for pinching off endocytic vesicles that is usually required for the classical clathrin-dependent endocytosis and for some non-classical pathways employed by viruses [38]. The pretreatment with dynasore, the dynamin inhibitor, resulted in a dose-dependent inhibition of DENV-2 production into both myeloid cell lines with maximum virus yield reductions about 99% in comparison with untreated infected cells (Fig 2C).

Finally, the effect on DENV-2 infection of the depletion of cholesterol by methyl-β-cyclodextrin was then analyzed. The compound altered the lipid raft/caveolae organization since the uptake of FITC-labelled cholera toxin, a marker of internalization through this route, was affected (Fig 1B). It was observed that the pretreatment with methyl-β-cyclodextrin had no influence on DENV-2 yield (Fig 2D), suggesting that the virus entry is caveolae-independent.

**Fig 3:**
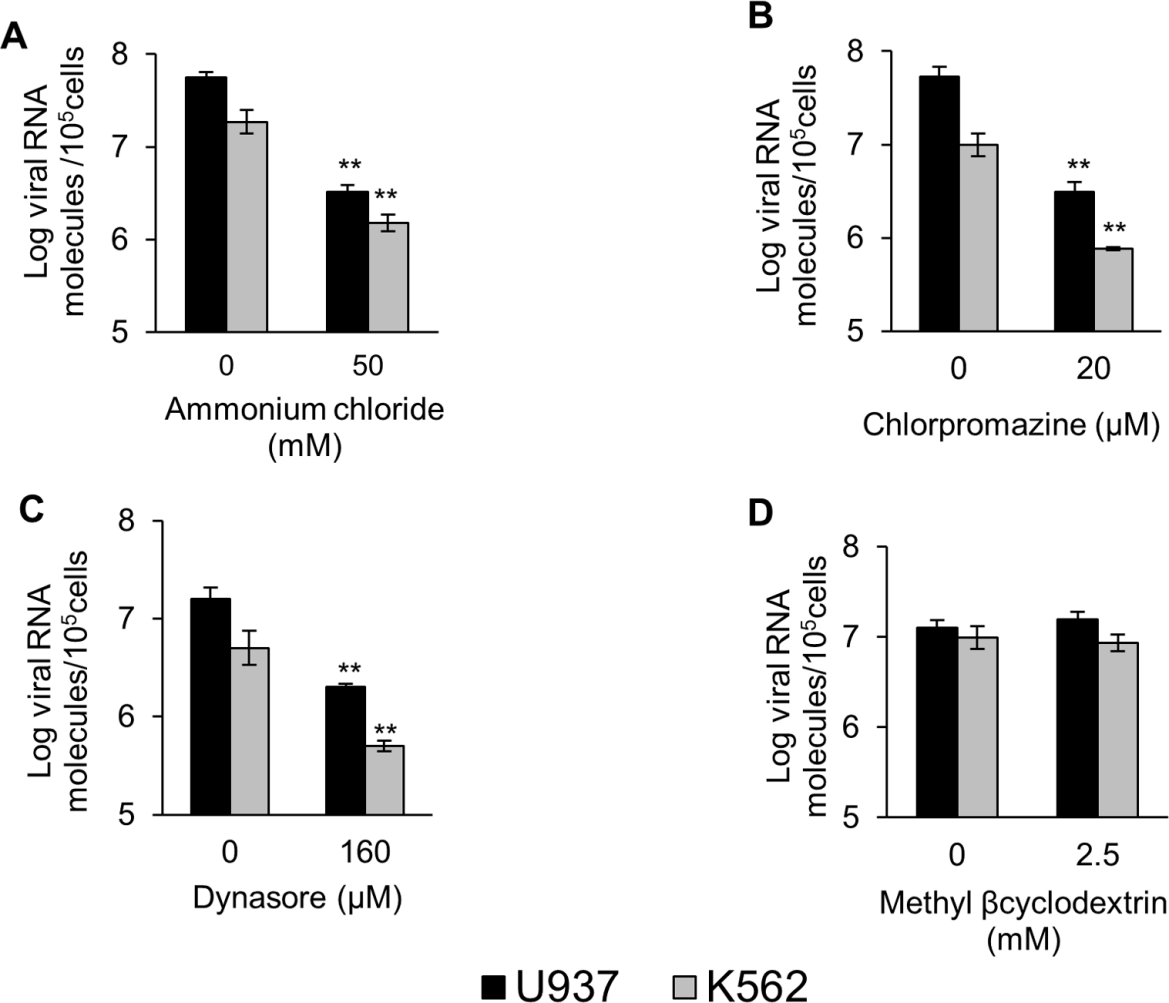
Effect of the endocytosis inhibitors on DENV-2 infection determined by quantitative RT-PCR. U937 (black bars) or K562 (grey bars) cells were treated with ammonium chloride (A), chlorpromazine (B), dynasore (C) or methyl-β-cyclodextrin (D), and then infected with DENV-2. At 12 h p.i., the total RNA was extracted from the cells with TRIzol and the amount of viral RNA molecules was measured by quantitative RT-PCR. Each bar is the mean of three independent experiments ± SEM. Asterisks indicate statistical significance between treated and control infected cells (** P < 0.0001).

The requirements for DENV-2 entry verified by virus yield reduction assay were further assessed determining the effect of the four inhibitors on the amount of synthesized viral RNA by quantitative RT-PCR. A significant reduction in the number of DENV-2 RNA molecules was also demonstrated after treatment with ammonium chloride, chlorpromazine or dynasore in both cells whereas no alteration was detected with methyl-β-cyclodextrin (Fig 3A–3D), confirming the infectivity data.

**Fig 4:**
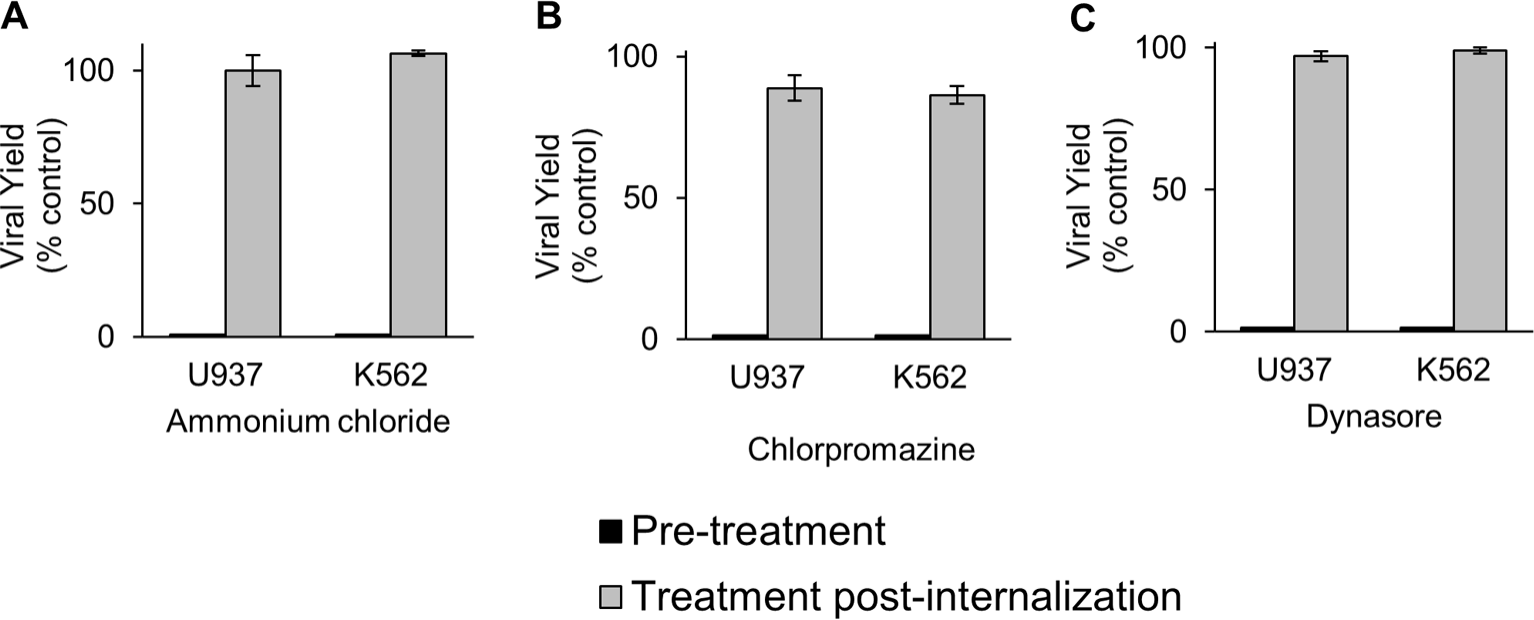
Time-dependence of the antiviral activity of the endocytic inhibitors. Cells were treated with 50 mM ammonium chloride (A), 20 μM chlorpromazine (B) or 160 μM dynasore (C) before infection with DENV-2 (pretreatment) or after 2 h of infection with DENV-2 (post internalization) at a m.o.i. of 5 PFU/cell. At 48 h p.i. the extracellular virus yields were determined by plaque formation in Vero cells and the results are expressed as % of virus yield in treated infected cells with respect to a control of infected cells without drug treatment. Each bar is the mean of three independent experiments ± SEM.

To further corroborate that the compounds only affected the early step of virus entry, cells were infected with DENV-2 under different treatment conditions. Cell cultures were treated with the inhibitors either after 2 h of infection with DENV-2, when virus penetration has occurred, or only by pretreatment. As expected a drastic reduction in virus yields was detected by drug treatment before infection whereas there was no inhibition when ammonium chloride, chlorpromazine or dynasore were added 2 h after infection (Fig 4A–4C), confirming that the compounds effectively blocked an early event during the DENV-2 entry process.

Thus, it can be concluded that the DENV-2 entry into myeloid U937 and K562 cells occurs by a low pH-, clathrin- and dynamin-dependent, caveola independent endocytic pathway and it can be effectively blocked by treatment with pharmacological inhibitors.

### The effect of the endocytic inhibitors on the Ab-mediated entry of DENV into human myeloid cells

To analyze the route of entry of DENV-2 in U937 and K562 cells in the presence of Ab and the effect of endocytic pharmacological inhibitors we employed the Ab 3H5 (IgG1 that binds to FcγRII and is reactive to DENV-2 E) and 2H2 (IgG2a that binds to both FcγRI and FcγRII and is reactive to prM of all DENV serotypes). Since U937 cells express both Fcγ-RI and Fcγ-RII whereas K562 cells express only Fcγ-RII, these systems available to establish ADE in DENV infection would allow discriminate the Ab-mediated entry route by FcγRI and FcγRII.

First, the conditions of in vitro enhancement of infection were established by incubation of 1.5 x 10^5^ PFU of DENV-2 with serial non neutralizing dilutions of the Ab 2H2 or 3H5 and subsequent infection of U937 or K562 cells with the mixtures. According to the previously obtained growth curves of DENV-2 in U937 and K562 cells (S3 Fig), the amount of virus employed for the mixtures did not produce detectable infective virus after infection of both cells in the absence of Ab. This was corroborated including in each experiment cells infected with the same amount of virus and without Ab as controls. At 72 h p.i., virus production was determined in cell supernatants by plaque formation in Vero cells. A 1:500 dilution of 3H5 or 2H2 Ab was chosen as adequate to produce a significant increase of infection in both cells (S4 Fig). To confirm that the observed increment in virus production was due to ADE, experiments were carried out using Ab to block the FcγRI or FcγRII receptors. The preincubation with aggregated IgG or with monoclonal Ab AT10 blocked the increase of virus yield triggered by 3H5 in both U937 and K562 cells, demonstrating that it is due to ADE and the entry is mediated by the FcγRII present in these cells (S4 Fig). The infection with DENV-2-2H2 was also blocked with aggregated IgG or AT10 in K562 cells that only express FcγRII, but in U937 cells the augmented infection with DENV-2-2H2 was blocked by soluble IgG, indicating that FcγRI is the receptor involved in the entry of this complex (S4 Fig). Although U937 cells express both receptors FcγRI and FcγRII, the detected binding of DENV-2-2H2 to FcγRI is in accordance with previous studies reporting the higher affinity of this IgG receptor [39]. Consequently, we decided to study the entry pathway of DENV-2 in the systems U937-DENV-2-2H2 and K562-DENV-2-3H5 to analyze the internalization predominantly through FcγRI or FcγRII, respectively.

**Fig 5:**
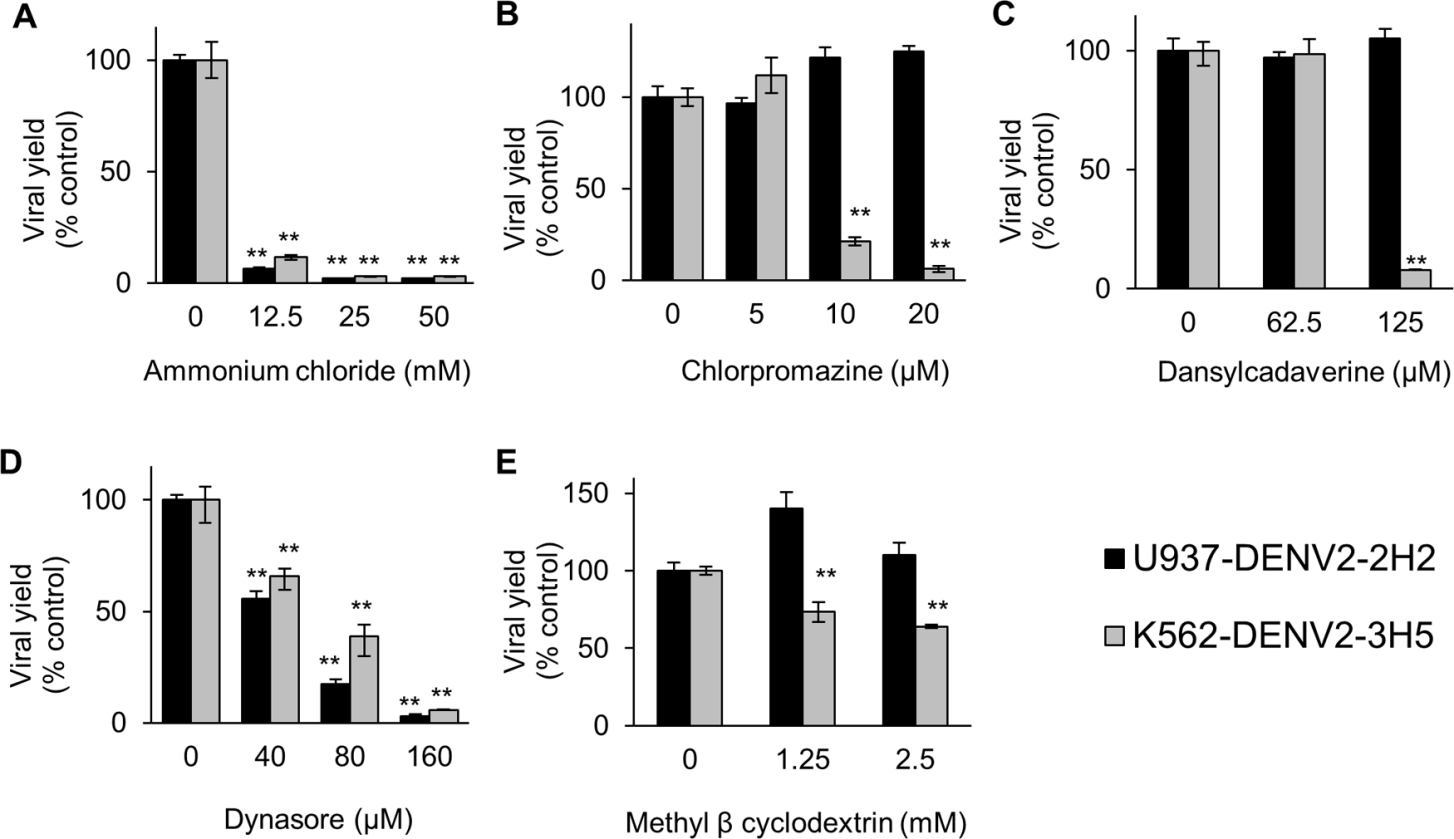
Effect of the endocytosis inhibitors on the Ab-mediated infection of U937and K562 cells. U937 (black bars) or K562 (grey bars) cells were treated with ammonium chloride (A), chlorpromazine (B), dansylcadaverine (C), dynasore (D) or methyl-β-cyclodextrin (E), and then infected with the complexes DENV-2-2H2 or DENV-2-3H5, respectively. At 72 h p.i., the virus yields were determined by plaque formation in Vero cells and the results are expressed as % of virus multiplication with respect to a control of complex infected cells without drug treatment. Each bar is the mean of three independent experiments ± SEM. Asterisks indicate statistical significance between treated and control infected cells (** P < 0.0001).

The requirement of acid pH for entry of both virus-Ab complexes, evaluated with ammonium chloride, was similar to that observed in Fig 2A for the infection at a higher m.o.i. in the absence of Ab. The inhibition in virus production was higher than 99% in both systems, either mediated by 2H2 or by 3H5 (Fig 5A), indicating that the Ab-mediated entry is acid pH-dependent.

The role of clathrin endocytosis was next tested using chlorpromazine. In K562 cells infected with the DENV-2-3H5 mixtures, a dose dependent inhibition of virus yield was observed after chlorpromazine treatment (Fig 5B) similarly as occurred in K562 cells infected in absence of Ab (Fig 2B). Surprisingly, chlorpromazine did not produce any inhibitory effect on the DENV-2-2H2 infection of U937 cells, even at the highest concentration of 20 μM (Fig 5B), contrasting with the strong reduction detected in U937 cells infected with DENV-2 in the absence of Ab (Fig 2B). This differential route of entry was further confirmed with dansylcadaverine, other pharmacological inhibitor used for blockade of clathrin-mediated endocytosis by affecting the clustering of membrane-bound ligands or particles in clathrin-coated pits [40,41]. The effect of dansylcadaverine on both systems was similar to that obtained with chlorpromazine (Fig 5C). Then, the complex DENV-2-3H5 appeared to be internalized in K562 cells driven by the Fcγ-RII in clathrin-coated vesicles and could be blocked by the clathrin-mediated endocytosis inhibitors whereas the internalization of DENV-2-2H2 in U937 cells mediated by Fcγ-RI was independent of clathrin and was not affected by these pharmacological inhibitors.

When the participation of dynamin in the Ab-mediated entry of DENV-2 was studied, a dose-dependent inhibitory activity of dynasore was observed in both cell systems (Fig 5D). Finally, the influence of the caveolae-dependent route on the Ab-mediated entry was analyzed by treatment with methyl-β-cyclodextrin. The infection of U937 cells with DENV-2-2H2 was not affected whereas a partial but significant reduction was detected in K562 cells infected with DENV-2-3H5 complexes, reaching about 36% inhibition in virus yield at a concentration of 2.5 mM (Fig 5E).

To further characterize the involvement of the Fc receptor in the differential utilization of clathrin-coated vesicles for the entry of DENV-2-Ab complex reported in Fig 5, the effect of chlorpromazine in the reversal systems, infection of U937 with DENV-2-3H5 and infection of K562 with DENV-2-2H2, was determined and compared with the infection with the virus-Ab complexes shown in Fig 5B. When U937 and K562 cells were treated with 20 μM chlorpromazine, DENV-2 production was inhibited more than 90% in the infection mediated either by 3H5 or 2H2 (Table 1). It can be concluded that the Ab-mediated entry of DENV-2 in K562 cells, that express only FcγRII, is dependent on clathrin-coated vesicles. In U937 cells, the DENV-2 entry mediated by 3H5, only recognized by FcγRII, also is clathrin-dependent as in K562 cells, whereas the infection mediated by 2H2, mainly recognized by FcγRI as shown in S3 Fig, occurs by an endocytic pathway independent of clathrin (Table 1).

**Table 1:** Effect of chlorpromazine on the Ab-mediated infection of U937 and K562 cells.

Virus-Ab complex	Virus yield (% inhibition ± SEM)	
	Cells K562	Cells U937
**DENV-2 – 3H5**	93.8 ± 1.2	99.4 ± 0.1
**DENV-2 – 2H2**	99.7 ± 0.1	0
**DENV-3 – 2H2**	84.6 ± 0.7	0

U937 or K562 cells were treated with 20 μM chlorpromazine and then infected with the indicated virus-Ab complex. At 72 h p.i., the virus yields were determined by plaque formation in Vero cells and the results are expressed as % of inhibition in virus yield with respect to a control of complex infected cells without drug treatment. Each value is the mean of three independent experiments ± SEM.

Studies on the primary entry of DENV in diverse mammalian cells have shown that virus enters usually by clathrin-mediated endocytosis, but the route of internalization via clathrin-dependence or not may be variable according to the virus serotype [14,19]. Then, we decided to test if the Ab-mediated entry by FcγRI in myeloid cells was also independent of clathrin for other DENV serotype. To this end, the experimental conditions for in vitro ADE infection in U937 and K562 cells were first established for the complex DENV-3-2H2 (S5 Fig) and then the effect of chlorpromazine was evaluated. The Ab 3H5 was not assayed in this system because it is not reactive with DENV-3. In the absence of Ab, the infection of myeloid cells with DENV-3 was previously reported to be inhibited by chlorpromazine in a dose-dependent manner, indicating a clathrin-mediated endocytic pathway [19], similarly as observed for DENV-2 (Fig 2B). Also as shown for DENV-2, chlorpromazine significantly inhibited virus yield from K562 cells infected with the complex DENV-3-2H2 whereas virus production from U937 cells infected with this complex was not affected when the clathrin pathway was arrested (Table 1), corroborating that the participation of the clathrin-dependent endocytic route for the Ab-mediated entry of DENV in myeloid cells appeared to be dependent on the involved FcγR.

## Discussion

After the initial transmission from the mosquito vector, the Fc-bearing cells of the mononuclear phagocyte lineage are crucial sites for virus propagation in the primary DENV infection as well as in secondary Ab-mediated infections, usually associated to the severe forms of dengue disease [3,42–46]. So far, specific antiviral agents against DENV are not available and supportive care is the only treatment for patients. Considering that the prevention of virus entry into these cells may be a convenient alternative for dengue therapy, we decided to study the mode of DENV internalization in two lines of myeloid cells in the absence and presence of Ab and evaluate the inhibitory activity of diverse biochemical inhibitors of endocytosis. Our results have shown that the DENV-2 entry into U937 and K562 cells in the absence of Ab is highly inhibited by early treatment with ammonium chloride, chlorpromazine and dynasore, but it is not affected by methyl-β-cyclodextrin (Figs 2, 3 and 4), indicating that DENV-2 utilizes a low pH-dependent, clathrin- and dynamin-mediated endocytic infectious pathway for the primary direct entry into both myeloid cells.

The need to be exposed at a intravesicular low pH for functional entry is shared by the four DENV serotypes in different mosquito and mammalian cells, including two studies in monocytic cells [22,24], in accordance with the conformational transition of E glycoprotein under acidic conditions that is known to trigger membrane fusion [35–37]. Then, the alkalinization of endosomes may prevent the low pH-induced fusion of viral envelope and endosomal membrane and block the DENV uncoating with release of the nucleocapsid to the cytoplasm. An approach for using this type of inhibitors was intended with the antimalarial agent chloroquine, a weak base known to affect intracellular endocytic pathways by increasing endosomal pH. This drug significantly reduced the virus production and the pro-inflammatory cytokines expression in DENV-2 infected cells [22]. However, two independent clinical trials performed in Vietnam and in Brazil showed the lack of effectiveness of chloroquine treatment in dengue fever patients [47,48].

The involvement of clathrin-mediated endocytosis, here shown by the potent antiviral activity of chlorpromazine against DENV-2 in U937 and K562 cells, was also demonstrated for DENV-2 entry in murine macrophage-like P338D1 cells with molecular inhibitors of the clathrin pathway [24] and in monocytes purified from human peripheral blood samples by using RNA interference silencing methods, that resulted in a marked reduction of DENV-2 infected cells and viral RNA load [21]. Chlorpromazine is a phenotiazine derivative in clinical use with antipsychotic effects [49]. In the line of repurposing for new indications clinically approved drugs, several pharmacologically approved phenotiazines were also found active entry inhibitors of hepatitis C virus, other member of Flaviviridae [50]. In particular, a significant antiviral activity against the DENV infection in human cell lines and in a mouse model was recently reported for prochlorperazine, a chlorpromazine analog [51], remarking the promising perspectives of this class of neuroleptic drugs in antiviral chemotherapy.

Regarding to the Ab-mediated entry of DENV, the internalization conducted by 3H5 or 2H2 Ab in myeloid cells was dependent on acid pH and dynamin as shown for Ab-independent infection (Fig 5). But a differential requirement of the clathrin-mediated endocytic route as determined by chlorpromazine treatment was observed for the entry of the DENV immune complexes depending on the FcγR involved in the complex uptake. The DENV-2 entry mediated by 3H5, an Ab only recognized by FcγRII, was dependent on clathrin-coated vesicles in both K562 and U937 cells, but when ADE was mediated by 2H2, that may be recognized by both FcγRI and FcγRII, the clathrin participation may vary according to the FcγR bound to the opsonized virus: the entry to K562 cells, mediated by FcRγII, was clathrin-dependent whereas the entry to U937 cells, here shown to be mediated by FcγRI, was clathrin-independent. FcγRI is a protein found exclusively in cells of the macrophage and dendritic cell lineages, with high affinity for monomeric IgG, whereas FcγRII is more broadly distributed in a variety of myelogenous cell types and with low affinity for monomeric IgG [52]. A similar response to chlorpromazine was observed for the internalization of the complex DENV-3-2H2 in myeloid cells (Table 1), denoting that this property was not serotype-specific.

As determined by methyl-β-cyclodextrin treatment, a differential requirement of cholesterol was observed depending on the FcγR involved: whereas the FcγRI-mediated entry was shown to be independent on cholesterol content, it seemed to be partially required for the endocytic mechanisms triggered by the virus-Ab complex binding to FcγRII (Fig 5). Similarly, it has also been shown that methyl-β-cyclodextrin abolished the infection of differentiated U937 cells with the DENV-4-Ab complex through FcγRII binding [23]. This receptor is a transmembrane glycoprotein known to translocate into the lipids rafts upon binding IgG-targets and it has been reported that the disruption of their structure by affecting the membrane cholesterol greatly inhibited the FcγRII-mediated IgG binding [53].

At present, we do not have an explanation for the dissimilar behaviour related to the FcγR directed entry in myeloid cells. Previous findings have described fundamental differences between FcγRI and FcγRII with respect to the influence of the signalling cascades following after binding of DENV-Ab complexes to myeloid cells: the abrogation of signalling competency was critical only for the FcγRI-mediated ADE but did not affect the FcγRII dependent DENV-Ab infection [54]. The authors proposed that the disparity between the immune-enhancing abilities of FcγRI and FcγRII could be related to different modes of DENV immune complex internalization between these FcγRs, as indicated by the experimental evidence provided in our studies. The use of alternative routes for the Ab-independent DENV entry has been previously reported in certain mammalian cells [14,15,19], thus it is not unexpected the possible coexistence of clathrin-dependent and independent pathways for the Ab-mediated DENV internalization in myeloid cells. Furthermore, it cannot be discarded that, besides the initial binding of the immune complexes to the FcγR, the use of other primary cell receptors may be still required to complete the complex penetration and infection process [11,55].

2H2 was characterized as a highly cross-reactive Ab that binds to immature particles of the four DENV serotypes by recognizing the precursor peptide prM [26,56]. After synthesis of viral macromolecules, the immature virions with projecting spikes formed by prM/E heterodimers are assembled in the endoplasmic reticulum. After passing the acidic trans-Golgi network, the viral particle undergoes a conformational change and cellular furin cleaves prM into M and pr, and finally pr is dissociated and the mature infective virions with a smooth envelope formed by homodimers of E covering the membrane-associated M protein are released. However, the cleavage of prM is not efficient and the infected cells secrete high levels of prM containing particles [57–59]. Then, the DENV suspensions consist of a mixture of fully infectious mature virions as well as fully immature particles containing prM and partially mature particles containing both prM and M proteins [60]. Although the immature particles are noninfective, it was shown that they become highly infectious in the presence of prM Ab that facilitates the binding and entry into FcR expressing cells [61–63]. Here, the prM-mediated ADE was confirmed and the efficiency was similar to the E-mediated ADE promoted by 3H5 Ab, remarking the importance of the abundant anti-prM Ab for the enhancement of human dengue disease [62]. Furthermore, the blockade with endocytic inhibitors was equally effective in the DENV-2H2- and DENV-3H5 infection when the FcγRII was involved in K562 cells, indicating that only the FcγR may alter the entry pathway and not the viral membrane protein bound to the Ab.

In conclusion, our studies have shown that the DENV entry into myeloid cells in the absence or presence of Ab can be blocked by diverse biochemical inhibitors affecting the cellular factors involved in endocytosis such as intravesicular pH, clathrin-coated vesicles and dynamin, although the presence of Ab can alter the entry pathway under certain conditions. A role of the FcγR for determining the route of internalization of the DENV-Ab complex was demonstrated, it remains to reveal the precise molecular basis of the differences detected among receptors in relation to the virus entry. The identification of specific virus-host interactions, such as those involved in the virus penetration, may allow the finding of host-targeted antivirals with wide spectrum of activity against those pathogenic flaviviruses with similar cell requirements for this process.

## Supporting information

S1 Fig Effect of the endocytosis inhibitors on the cell viability. U937 (black bars) or K562 (grey bars) cells were treated with serial concentrations of ammonium chloride (A), chlorpromazine (B), dansylcadaverine (C), dynasore (D) or methyl-β-cyclodextrin (E). After 3 h of incubation at 37°C, the viable cells were counted by trypan blue exclusion. The results are expressed as % of cell viability in treated cultures with respect to a control of cells without drug treatment. Each bar is the mean of three independent experiments ± SEM.
(TIF)

S2 Fig Effect of the endocytosis inhibitors on virion viability. DENV-2 suspensions containing 1x10^6^ PFU/ml were incubated with different concentrations of ammonium chloride (A), chlorpromazine (B), dansylcadaverine (C) or dynasore (D) for 2 h at 37°C. Then, samples were filtered through cellulose membranes to eliminate free drug and the residual infectivity was determined by plaque formation. Each bar is the mean of three independent experiments ± SEM.
(TIF)

S3 Fig Growth curves of DENV-2 in U937 and K562 cells. Cultures of U937 (A) or K562 (B) cells were infected with DENV-2 at the indicated m.o.i. and incubated at 37°C. At different post-infection times extracellular virus yields were determined by a plaque assay. Each bar is the mean of three independent experiments ± SEM.
(TIF)

S4 Fig Establishment of an in vitro ADE model with DENV-2. A-B. DENV-2 suspensions containing 1.5x10^5^ PFU were incubated with different dilutions of 2H2 (A) or 3H5 (B) Ab during 1 h at 37°C. Then, U937 or K562 cells were infected with the mixtures and at 72 h p.i. the virus yields were determined by plaque formation in Vero cells. C-D. U937 or K562 cells were incubated with 30 μg/ml of Ab AT10, soluble or aggregated human IgG during 30 min at 4°C. After washing, the cells were infected with the mixtures DENV-2-2H2 (C) or DENV-2-3H5 (D). The viral yields were determined at 72 h p.i. by plaque formation in Vero cells. Each value is the mean of three independent experiments ± SEM.
(TIF)

S5 Fig Establishment of an in vitro ADE model with DENV-3. A. DENV-3 suspensions containing 1.5x10^5^ PFU were incubated with different dilutions of 2H2 during 1 h at 37°C. Then, U937 or K562 cells were infected with the mixtures and at 72 h p.i. the virus yields were determined by plaque formation in Vero cells. B. U937 or K562 cells were incubated with 30 μg/ml of Ab AT10, soluble or aggregated human IgG during 30 min at 4°C. After washing, the cells were infected with DENV-3-2H2. The viral yields were determined at 72 h p.i. by plaque formation in Vero cells. Each bar is the mean of three independent experiments ± SEM.
(TIF)
