# Supplementary figures and images for: Blockade of dengue virus entry into myeloid cells by endocytic inhibitors in the presence or absence of antibodies
Source: PLoS Negl Trop Dis. 2018 Aug 9;12(8):e0006685. doi: 10.1371/journal.pntd.0006685 (PMC6103515; doi:10.1371/journal.pntd.0006685)

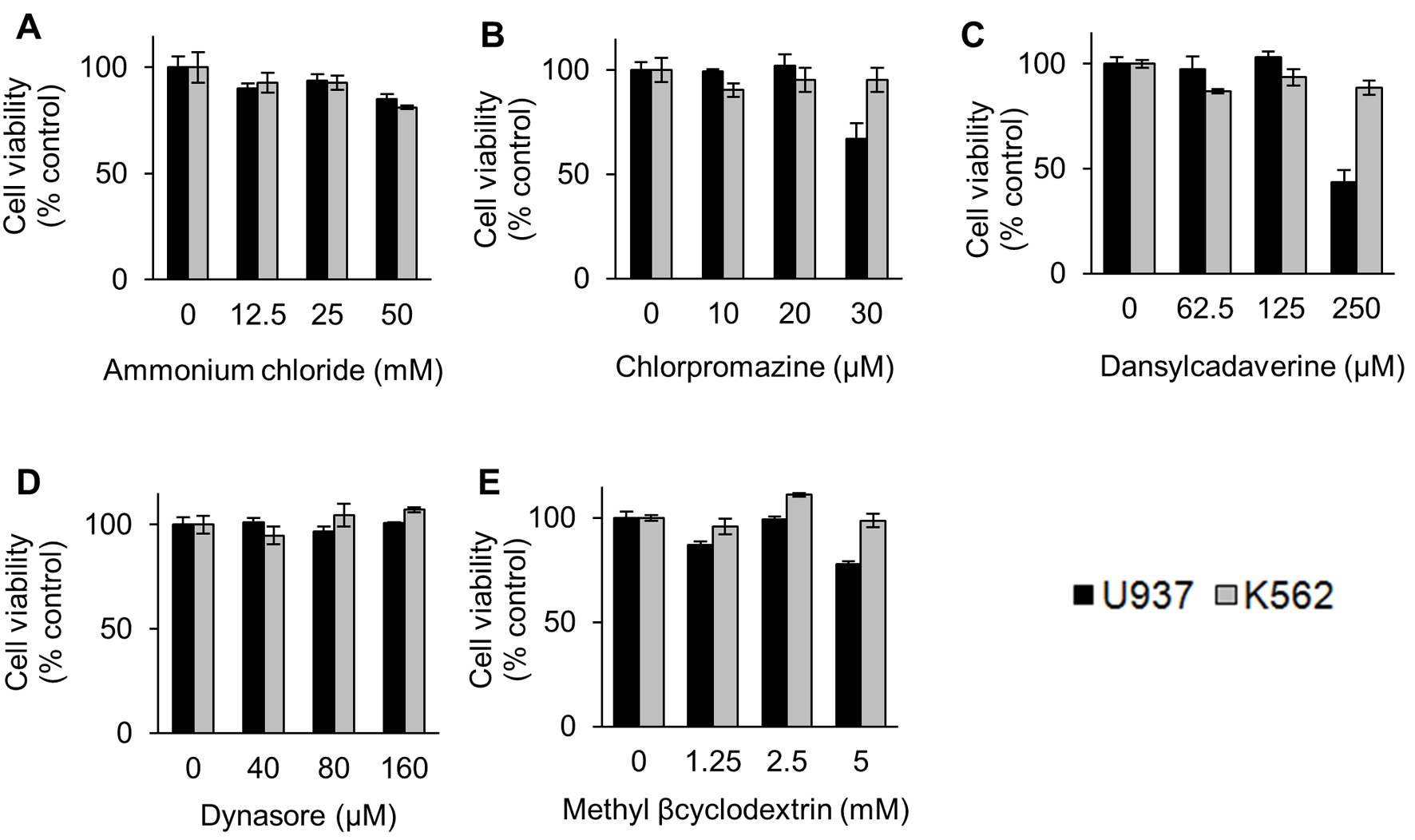

Supplement: S1 Fig — U937 (black bars) or K562 (grey bars) cells were treated with serial concentrations of ammonium chloride (A), chlorpromazine (B), dansylcadaverine (C), dynasore (D) or methyl-β-cyclodextrin (E). After 3 h of incubation at 37°C, the viable cells were counted by trypan blue exclusion. The results are expressed as % of cell viability in treated cultures with respect to a control of cells without drug treatment. Each bar is the mean of three independent experiments ± SEM. (TIF) [file pntd.0006685.s001.TIF]

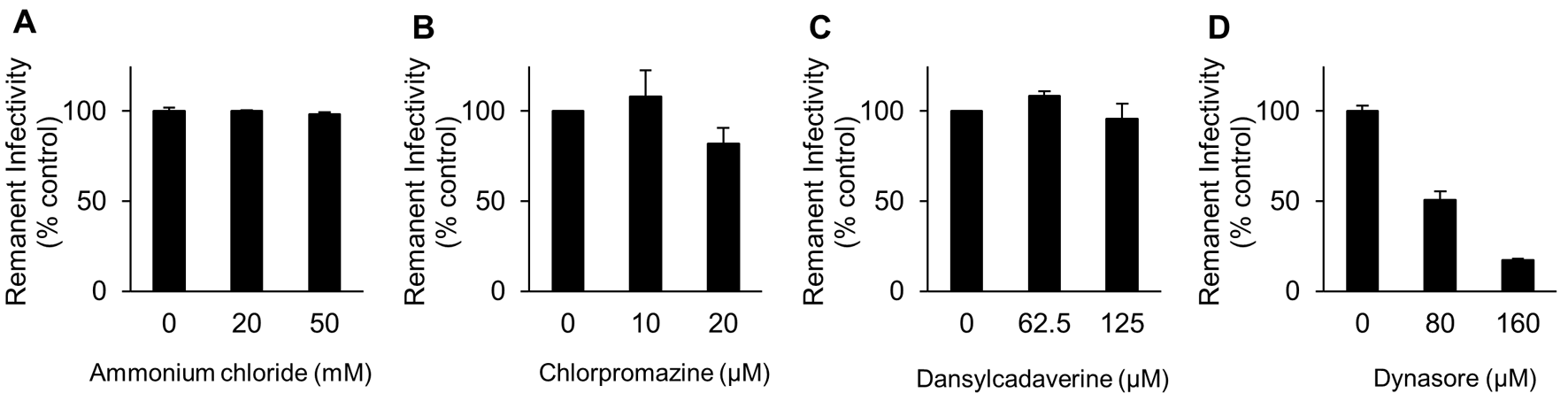

Supplement: S2 Fig — DENV-2 suspensions containing 1x10^6^ PFU/ml were incubated with different concentrations of ammonium chloride (A), chlorpromazine (B), dansylcadaverine (C) or dynasore (D) for 2 h at 37°C. Then, samples were filtered through cellulose membranes to eliminate free drug and the residual infectivity was determined by plaque formation. Each bar is the mean of three independent experiments ± SEM. (TIF) [file pntd.0006685.s002.TIF]

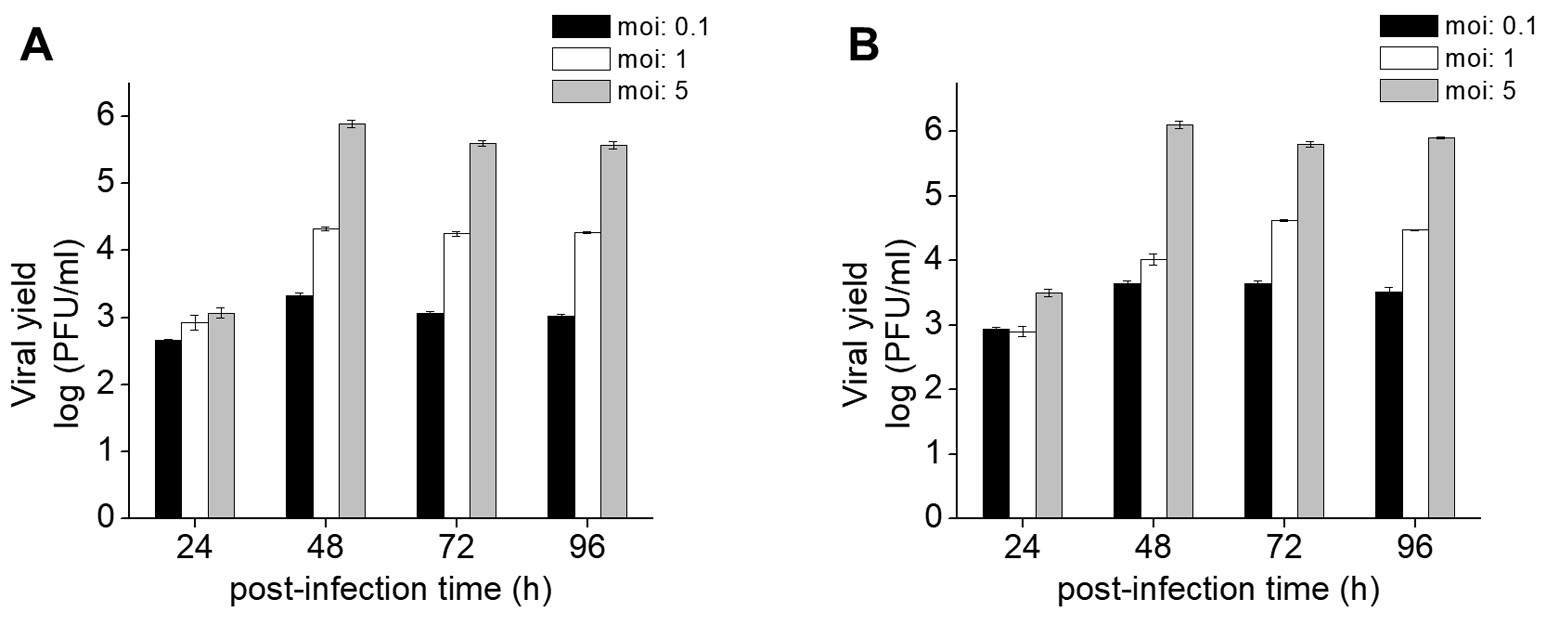

Supplement: S3 Fig — Cultures of U937 (A) or K562 (B) cells were infected with DENV-2 at the indicated m.o.i. and incubated at 37°C. At different post-infection times extracellular virus yields were determined by a plaque assay. Each bar is the mean of three independent experiments ± SEM. (TIF) [file pntd.0006685.s003.TIF]

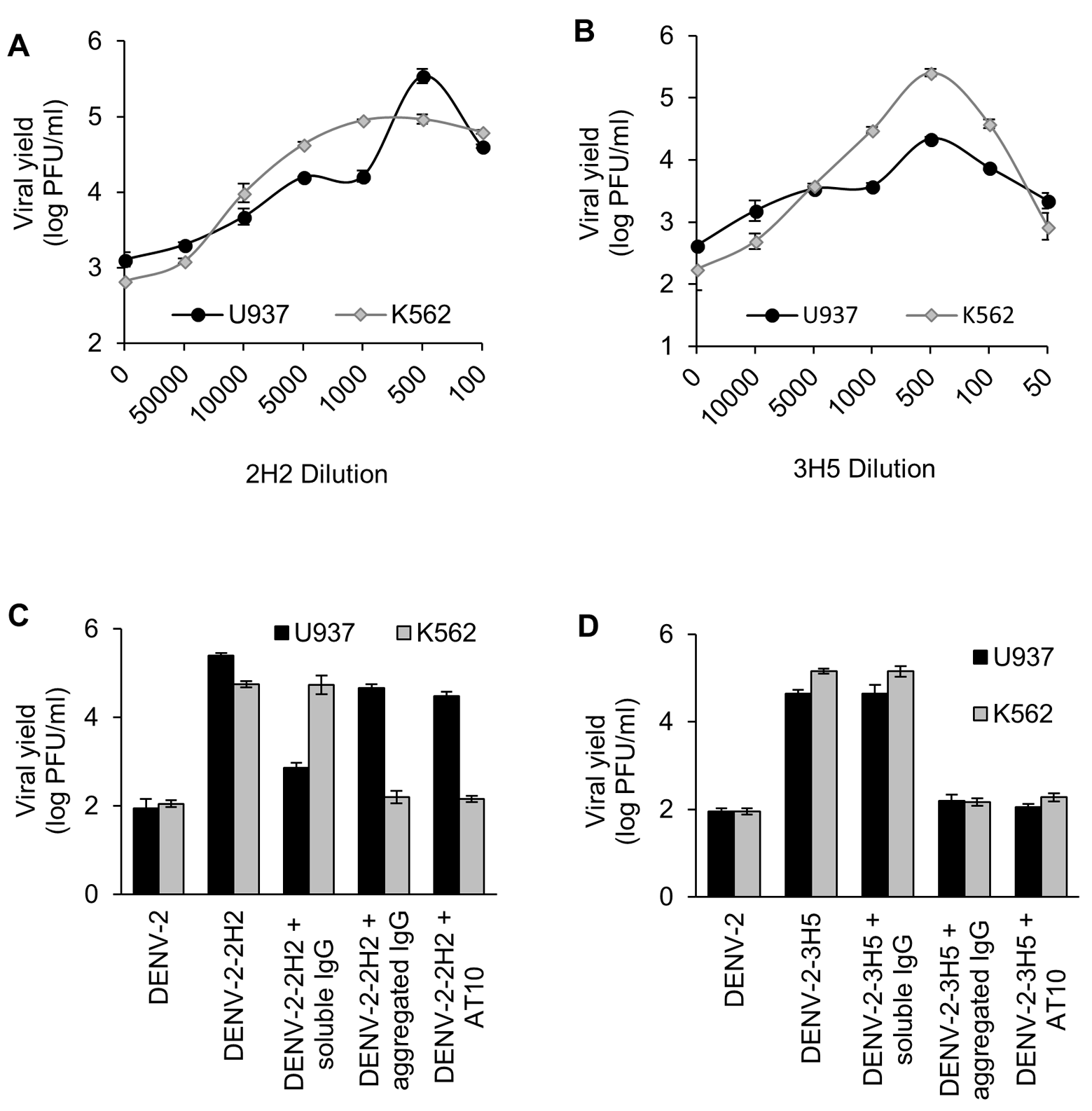

Supplement: S4 Fig — A-B. DENV-2 suspensions containing 1.5x10^5^ PFU were incubated with different dilutions of 2H2 (A) or 3H5 (B) Ab during 1 h at 37°C. Then, U937 or K562 cells were infected with the mixtures and at 72 h p.i. the virus yields were determined by plaque formation in Vero cells. C-D. U937 or K562 cells were incubated with 30 μg/ml of Ab AT10, soluble or aggregated human IgG during 30 min at 4°C. After washing, the cells were infected with the mixtures DENV-2-2H2 (C) or DENV-2-3H5 (D). The viral yields were determined at 72 h p.i. by plaque formation in Vero cells. Each value is the mean of three independent experiments ± SEM. (TIF) [file pntd.0006685.s004.TIF]

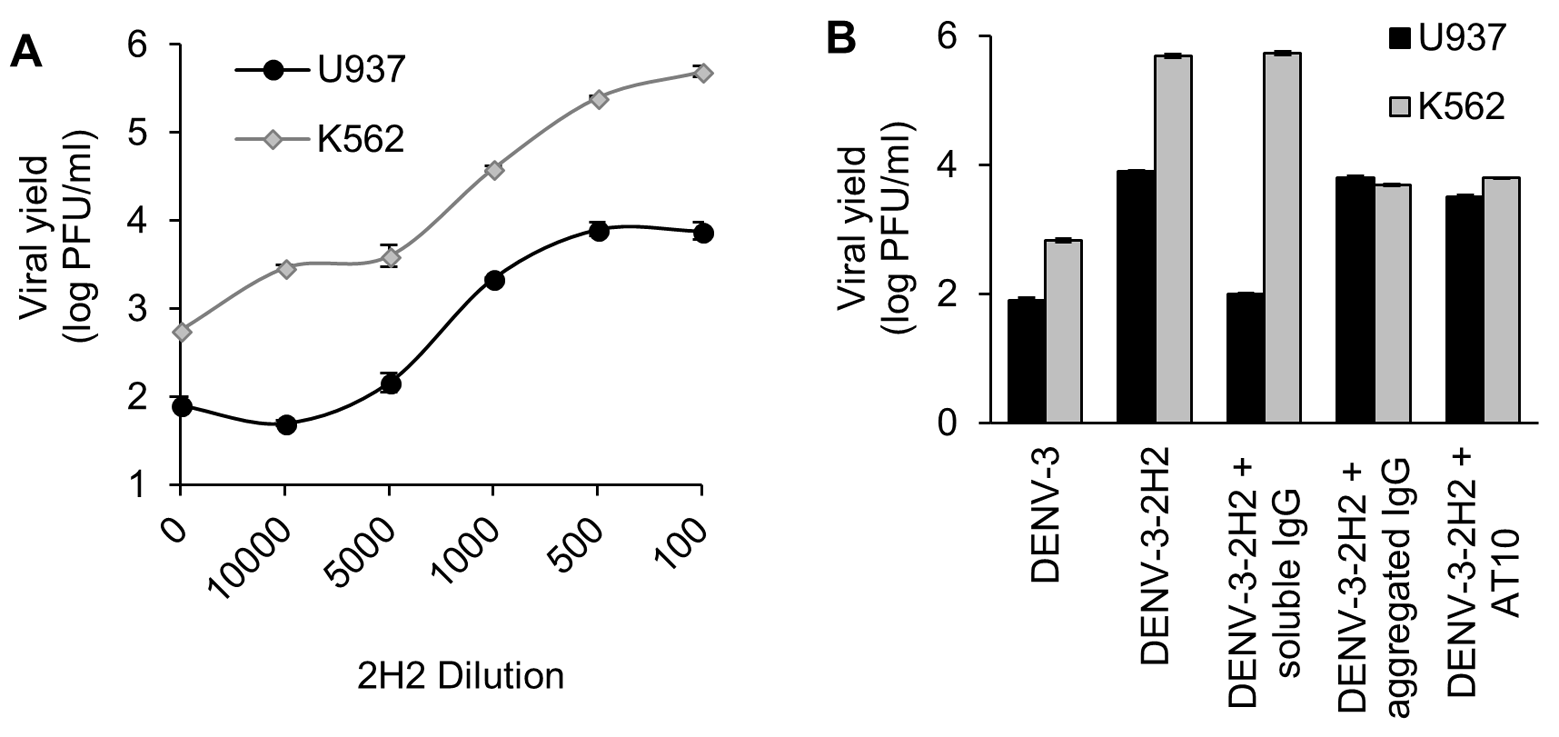

Supplement: S5 Fig — A. DENV-3 suspensions containing 1.5x10^5^ PFU were incubated with different dilutions of 2H2 during 1 h at 37°C. Then, U937 or K562 cells were infected with the mixtures and at 72 h p.i. the virus yields were determined by plaque formation in Vero cells. B. U937 or K562 cells were incubated with 30 μg/ml of Ab AT10, soluble or aggregated human IgG during 30 min at 4°C. After washing, the cells were infected with DENV-3-2H2. The viral yields were determined at 72 h p.i. by plaque formation in Vero cells. Each bar is the mean of three independent experiments ± SEM. (TIF) [file pntd.0006685.s005.TIF]
